# Exploring marine-derived compounds as potential anticancer agents: Mechanisms and therapeutic implications

**DOI:** 10.1016/j.cpt.2025.08.004

**Published:** 2025-08-26

**Authors:** Nagaraju Bandaru, Yash Pramod Patil, Sumit Dilip Ekghara, Kunal Sharad patil, Mohan Gandhi Bonthu

**Affiliations:** a Department of Pharmacology, School of Pharmaceutical Sciences, Sandip University, Nashik, Maharashtra, 422213, India; b Department of Pharmaceutical Analysis and Quality Assurance, School of Pharmaceutical Sciences, Sandip University, Nashik, Maharashtra, 422213, India

**Keywords:** Marine products, Antineoplastic agents, Apoptosis, Angiogenesis inhibitors

## Abstract

Marine-derived compounds have emerged as a promising frontier in cancer research owing their remarkable structural diversity and broad-spectrum bioactivities. The marine environment, harboring diverse organisms (e.g., sponges, algae, tunicates, mollusks, and marine microbes), is a prolific source of novel bioactive molecules with potent anticancer activities. Key classes of these compounds include alkaloids, polysaccharides, peptides, terpenoids, and polyketides, which exert antitumor effects through diverse mechanisms, including the induction of apoptosis, inhibition of angiogenesis, modulation of immune responses, interference with cell cycle progression, and targeting of critical signaling pathways involved in tumorigenesis and metastasis. Notably, marine-derived drugs such as trabectedin, eribulin, and plitidepsin have received regulatory approval for the treatment of various malignancies, demonstrating the translational potential of these natural compounds. Ongoing clinical and preclinical investigations are exploring a wide range of marine metabolites for their cytotoxic, anti-proliferative, and chemosensitizing properties. Advances in marine biotechnology, including genome mining, synthetic biology, and fermentation technologies, have significantly facilitated the discovery, sustainable production, and structural optimization of marine natural products. However, challenges such as low yield, structural complexity, limited water solubility, and poor bioavailability hinder their broader clinical application. The integration of novel drug delivery systems, such as nanoparticles, liposomes, and conjugates, offers a viable solution to overcome these limitations and improve pharmacokinetic profiles. This review provides a comprehensive overview of the mechanisms of action, therapeutic applications, and clinical development of marine-derived anticancer compounds. It also emphasizes the need for deeper insights into their molecular targets and the potential for synergistic use with existing chemotherapeutic agents. Future directions should focus on exploring untapped marine biodiversity, developing eco-friendly harvesting strategies, and developing innovative delivery platforms to fully harness the therapeutic promise of the marine pharmacopeia in oncology.

## Highlights

• Marine ecosystems are rich in unique compounds such as alkaloids, peptides, polysaccharides, and terpenes with potent anticancer activities.
• Marine-derived compounds exert their effects through multiple mechanisms, including apoptosis induction, cell cycle arrest, angiogenesis inhibition, and metastasis suppression.
• Several marine compounds, like trabectedin and eribulin, have progressed to clinical use, showcasing the translational potential of marine-derived molecules in cancer therapy.
• Despite promising results, challenges such as sustainable sourcing, structural complexity, and optimization for clinical application remain key hurdles in marine drug development.

## Graphical abstract

It represents the leading sources of marine-derived compounds, prevention/treatment strategies, and how marine drugs target cancer cells, key benefits include fewer side effects, potential for combined therapies, novel mechanisms of action, reduced drug resistance, inhibition of tumor growth and metastasis, enhanced immune system function, and targeted action on cancer cells, the image also details methods by which marine drugs treat cancer, including inducing apoptosis, inhibiting cell division, targeting specific cancer pathways, inhibiting angiogenesis, preventing metastasis, inhibiting cancer stem cells, chemotherapy sensitization, and disrupting DNA repair replication in cancer cells.

**Figure:**
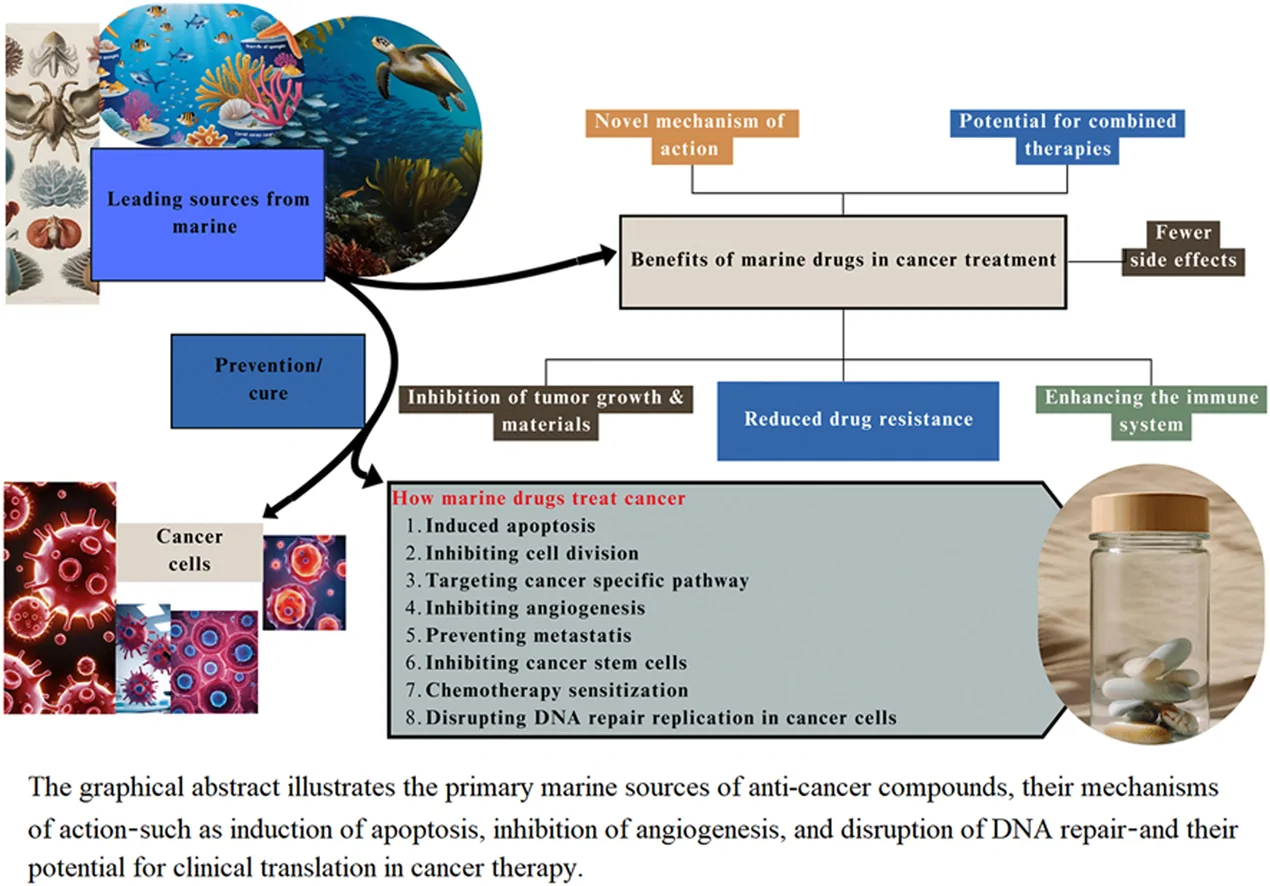

## Introduction

### The global cancer burden

Cancer remains one of the most pressing global public health challenges, accounting for one of the leading causes of death worldwide. According to the Global Cancer Observatory (GLOBOCAN) 2022 data from the World Health Organization (WHO), there were an estimated 20 million new cancer cases and nearly 10 million deaths globally in 2022 alone. Projections indicate that the annual incidence may exceed 28 million by 2040, driven by demographic shifts, lifestyle changes, and environmental factors. This escalating burden not only threatens human health but also imposes immense socioeconomic stress, particularly on healthcare systems in low- and middle-income countries, where access to diagnostic and treatment facilities remains limited.

### The need for novel therapies

Despite remarkable advances in cancer diagnosis and treatment, current therapeutic strategies such as chemotherapy, radiation therapy, targeted therapy, and immunotherapy face several critical limitations. These include drug resistance, high systemic toxicity, off-target effects, and limited efficacy against certain aggressive or refractory cancers. Moreover, tumor heterogeneity and the dynamic nature of cancer evolution often hinder the long-term effectiveness of conventional treatments. These challenges highlight the urgent need to identify and develop novel, effective, and less toxic anticancer agents that can overcome resistance and improve clinical outcomes.^1^

### The role of natural products in cancer drug discovery

Natural products have historically served as an indispensable source of therapeutic agents, particularly in oncology. Over 50% of current anticancer drugs are derived from or inspired by natural sources.^2^ In recent decades, the marine environment has gained attention as a largely untapped reservoir of structurally diverse and biologically potent compounds. Marine organisms, including sponges, tunicates, algae, mollusks, and marine-derived microorganisms, produce a wide array of secondary metabolites with unique chemical architectures that are rarely found in terrestrial sources. These compounds exhibit promising bioactivities, including cytotoxic, antiproliferative, pro-apoptotic, anti-angiogenic, and immunomodulatory effects, making them desirable candidates for anticancer drug development.^3^

### Successful marine-derived examples

Several clinically approved anticancer agents exemplify the translational potential of marine-derived compounds. Cytarabine (Ara-C), derived from the Caribbean sponge Cryptotethya crypta, was among the first marine-derived drugs approved for the treatment of hematologic malignancies.^4^ Trabectedin, isolated from the sea squirt Ecteinascidia turbinata, is approved for soft tissue sarcoma and ovarian cancer. Eribulin mesylate, a synthetic analog of halichondrin B from the sponge *Halichondria okadai*, is used in metastatic breast cancer. Brentuximab vedotin, an antibody–drug conjugate that utilizes the marine-derived toxin monomethyl auristatin E (MMAE), has succeeded in Hodgkin and anaplastic large cell lymphoma. These successes affirm marine natural products' clinical relevance and promise in modern oncology.^5^

This review aims to systematically explore marine-derived compounds with demonstrated anticancer activity, focusing on their molecular mechanisms of action, such as induction of apoptosis, cell cycle arrest, inhibition of angiogenesis, and modulation of cancer signaling pathways. Furthermore, the review will evaluate these compounds' therapeutic potential and translational relevance, highlighting current challenges and future directions in marine pharmacology. By synthesizing current knowledge, we seek to provide a comprehensive framework for advancing marine natural products as viable anticancer agents.

## Various types of cancer

### Leukemia

**Figure 1:**
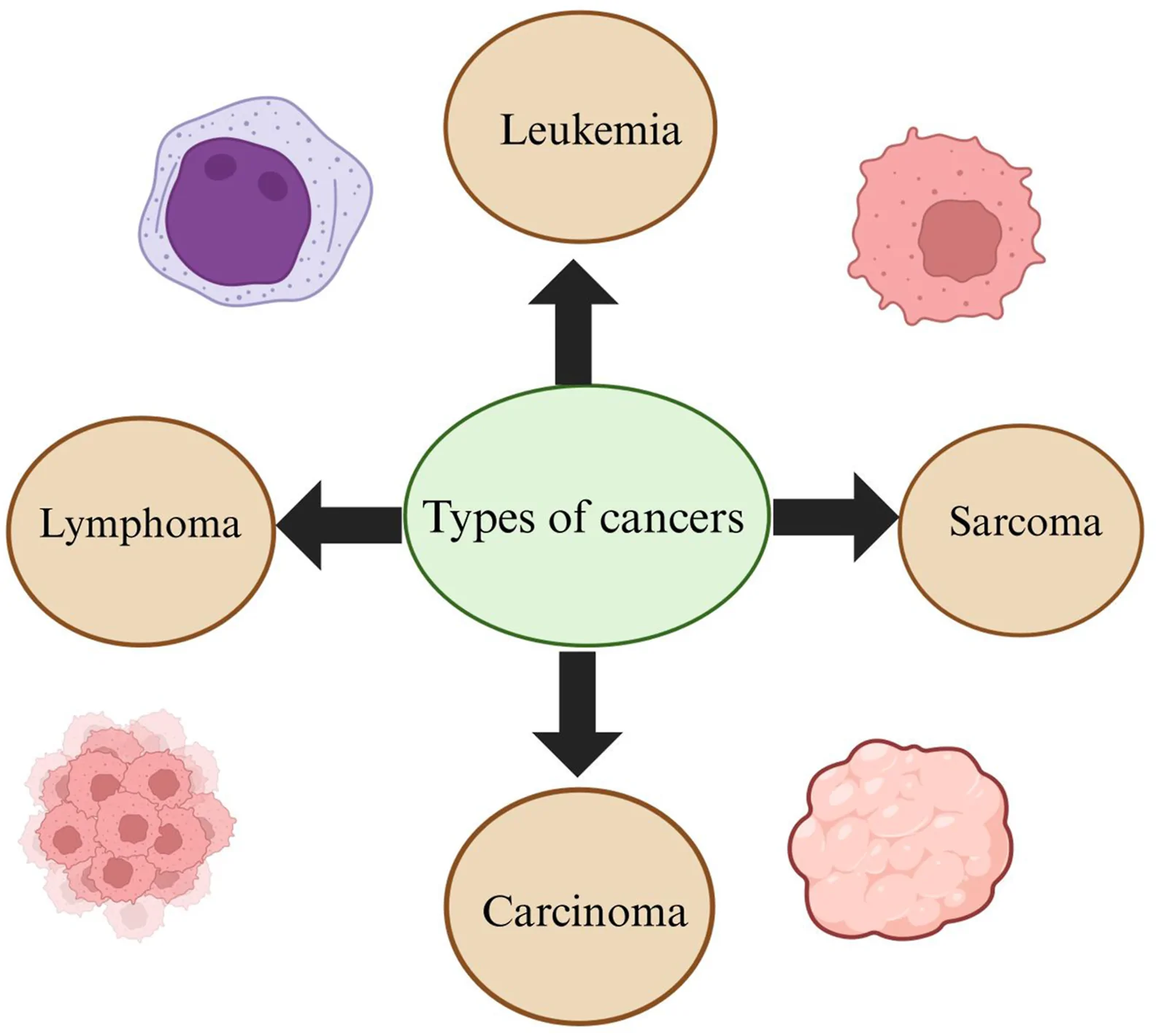
Classification of major cancer types based on cell lineage and progression.

The development of aberrant leukocytes determines whether leukemia is classified as primary or secondary. The rate of disease progression determines whether leukemia is acute or chronic. and their cell of origin classifies them as myeloid or lymphoid. The myeloid lineage is implicated in the most prevalent subtypes, which include acute myeloid leukemia (AML) and chronic myeloid leukemia (CML). In contrast, the lymphoid lineage gives rise to acute lymphoblastic leukemia (ALL) and chronic lymphocytic leukemia (CLL). Leukemia is associated with NK cells, mature B-cells, and T-cells leukemia, which are less common types of mature white blood cells. Nonetheless, the following the development of next-generation sequencing (NGS) and the identification of many biomarkers in 2016, various changes were made to align with the World Health Organization's (WHO) standard classification of acute leukemia, also known as myeloid neoplasms [Figure 1].^6^ Global Cancer Observatory (GLOBOCAN), a worldwide observer for cancer trends, reported 474,519 cases worldwide, with 67,784 in North America. Age-standardized incidence rates are approximately 3.2 per 100,000 population, and age-standardized mortality rates are approximately 11 per 100,000 population.^8^ Due to inexperience, it lowers the extremely early mortality linked to a nonleukemia environment. It improves long-term results by meaningfully changing the diagnosis in 30–40 percent of patients.

During the 4- to 8-week induction therapy phase, the mortality rate has decreased from 10%–40% to 2%–5%. It provides cutting-edge treatments on experimental protocols that, one to two decades later, frequently become the new norms of care.^8-^

These are broadly categorized based on the cell lineage involved—lymphoid or myeloid—and the pace of disease progression—acute or chronic. ALL is most commonly seen in children but can also occur in adults. It originates from lymphoid precursor cells and progresses rapidly, necessitating urgent treatment. In contrast, AML primarily affects adults and arises from myeloid precursor cells. Like ALL, it is fast-growing and accumulates immature myeloid blasts in the bone marrow, causing bone marrow failure. On the other hand, CML, typically diagnosed in middle-aged to older adults, is associated with a specific genetic abnormality—the Philadelphia chromosome (*BCR-ABL* fusion gene)—which results in continuous activation of tyrosine kinase. This leads to a slower but progressive disease course that can be effectively managed with targeted therapy such as imatinib.^9^

### Lymphoma

Lymphomas affecting the lymphatic system are classified into two major types: Hodgkin's Lymphoma (HL) and Non-Hodgkin Lymphoma (NHL). HL is characterized by the presence of Reed-Sternberg cells, large abnormal B lymphocytes as a hallmark of the disease. It commonly affects young adults and tends to spread in a predictable manner from one group of lymph nodes to another. With modern treatment regimens, HL has a high cure rate. In contrast, NHL comprises diverse lymphoid cancers arising from B cells, T cells, or natural killer (NK) cells. It encompasses both indolent (slow-growing) and aggressive forms, such as follicular lymphoma and diffuse large B-cell lymphoma. NHL is more heterogeneous in its presentation, biological behavior, and prognosis compared to HL, and treatment strategies are tailored based on the specific subtype and stage.^10^

### Carcinoma

Squamous cell carcinoma, which is the second most frequent category of tumor on the skin in the United States, is a serious public health concern since its prevalence is increasing year after year. Squamous cell carcinoma is the second most frequent sort of skin cancer in the U.S. Risk factors include male gender, advanced age, fair skin, chronic wounds, immunosuppression, and certain hereditary illnesses. Environmental factors include ultraviolet light and a history of squamous cell cancer. The lymph nodes are the most frequent location of metastasis, even though it is uncommon. Timely surveillance, early diagnosis, and fast treatment are crucial to reducing the risks of morbidity and mortality as the frequency continues to rise, raising serious public health concerns. Regular full-body skin examinations and photoprotection are advised. Although surgical excision is the primary treatment for most cases, new therapeutic approaches are continually emerging. In more severe situations, radiation therapy and systemic oncologic can be necessary.^11^

Skin cancer is the most prevalent form of cancer worldwide. Basal cell carcinoma (BCC) and squamous cell carcinoma (SCC) are the most common non-melanoma skin cancers among its major types. BCC originates from the basal cells of the epidermis and is the most frequently diagnosed skin cancer. It typically exhibits a slow growth rate and rarely metastasizes, but can cause significant local tissue destruction if left untreated. SCC arises from squamous keratinocytes and has a higher potential for local invasion and metastasis compared to BCC. Both types are strongly associated with chronic ultraviolet (UV) radiation exposure, and their incidence continues to rise globally. Including BCC alongside SCC is important for a complete and accurate discussion of skin cancers, especially given BCC's predominance in clinical practice.^12^

### Sarcoma

Soft tissue sarcomas (STS) are a collection of over 60 neoplasms that can develop in any part of the body and affect people of every age. Tumors like these can be caused by skeletal muscle, adipose tissue, blood, lymphatics, connective tissues, or peripheral nerves. They can also be aggressive metastatic angiosarcomas or benign lipomas, among other kinds.^13^ Non-neoplastic diseases are very difficult to distinguish from STS since they frequently mimic STS. Soft tissue sarcomas fall into three categories: trunk, extremities, and retroperitoneal. Most cases of STS happen on their own. However, there is evidence linking environmental exposures, radiation, and germline alterations.^14^

## Common forms of cancer

### Breast cancer

**Figure 2:**
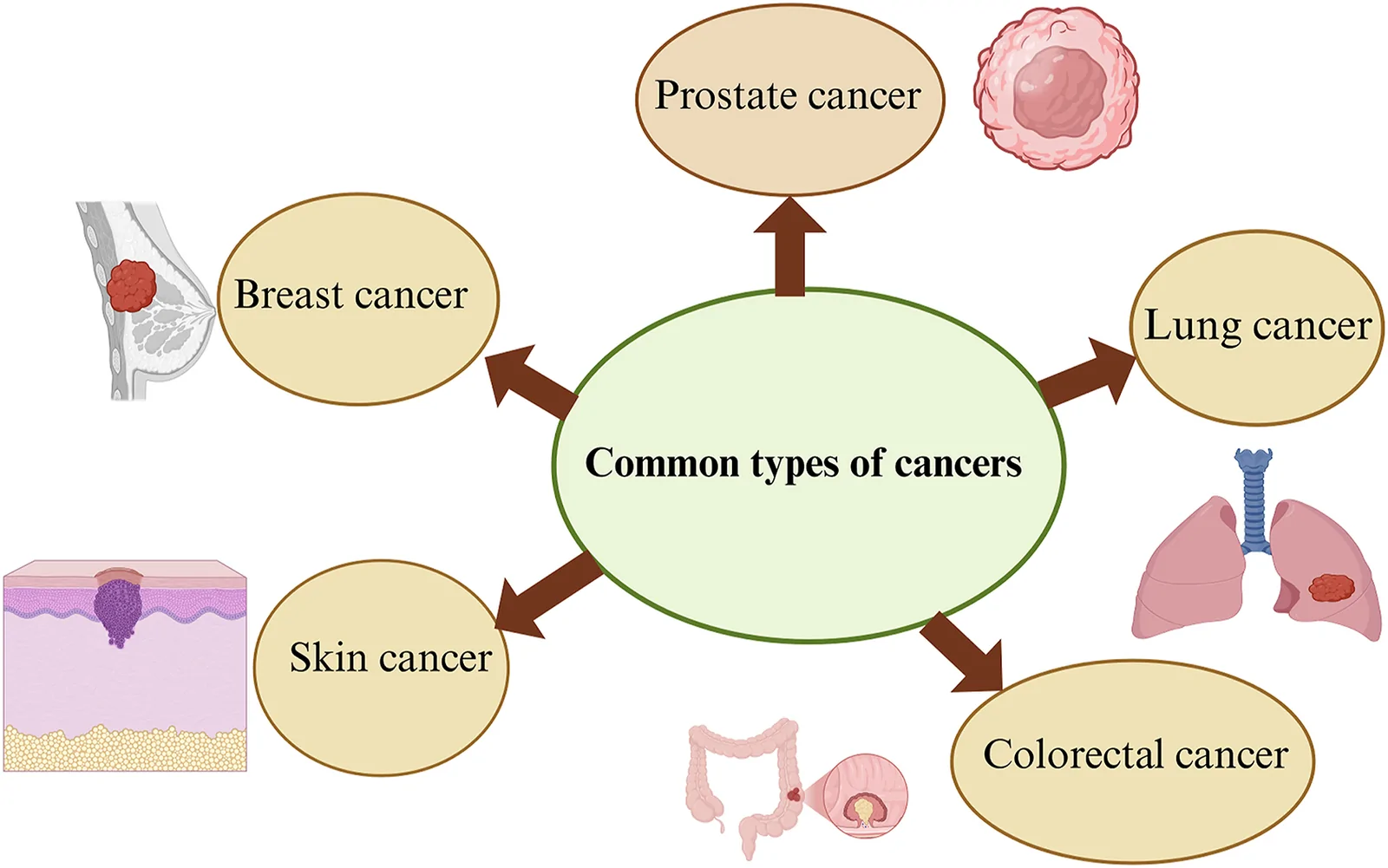
Common types of cancer.

Cancer is the second leading cause of mortality among women. Breast cancer is the most prevalent type of cancer diagnosed in women worldwide.^15^ Situated superficial to the pectoralis major muscle, the breasts are a pair of glands that vary in size and density. They include milk-roducing lobules; these lobules are often clustered together to create lobes that are dotted with fat. Milk and other fluids produced in acini are released by lactiferous ducts that emerge at the nipple. By attaching the breast to the underlying muscular tissue, Cooper ligaments provide stability [Figure 2].^16^ The two main types of breast cancer are defined by their origin: ductal carcinoma develops in the ductal epithelium, whereas lobular carcinoma develops in the lobules. Ductal carcinoma is the most prevalent form. Numerous identified risk factors exist for breast cancer. Instead of relying on symptoms, screening programs have been successful in detecting the majority of breast tumors in Western countries. However, in most of the poor world, the first indication is often a breast lump or odd nipple discharge.^17^ Breast cancers are diagnosed by physical assessment, tissue biopsy, and breast imaging. Hormone therapy, radiation, chemotherapy, surgery, and, more recently, immunotherapy are all viable treatment choices. Personalized therapy selections are influenced by characteristics such as tumor markers, stage, histology, and hereditary disorders.

### Prostate cancer

In 2018, prostate cancer accounted for 1,276,106 new cases and 358,989 deaths (3.8% of all male cancer-related deaths), making it the second most frequent cancer in men worldwide, after lung cancer.^28^ The prevalence and mortality rates of prostate cancer are rising globally, with an average diagnosis age of 66 years. In addition, African-American men are roughly twice as likely to die from the disease as white men, and the mortality estimates are more substantial—158.3 new cases are identified for every 100,000 men.^18^

### Lung cancer

Lung cancer is a genetically heterogeneous disease, and advancements in molecular oncology have revealed that specific genetic mutations play a critical role in driving tumor initiation, progression, and therapeutic response. Among these, mutations in the epidermal growth factor receptor (*EGFR*) gene are commonly found in non-small cell lung cancer (NSCLC), particularly in non-smokers and East Asian populations. *EGFR* mutations lead to constitutive activation of downstream signaling pathways such as the Phosphoinositide 3-Kinase/Protein Kinase B (PI3K/AKT) pathway and Mitogen-Activated Protein Kinase (MAPK), promoting uncontrolled cell proliferation and survival. This discovery has revolutionized lung cancer treatment, with EGFR tyrosine kinase inhibitors (TKIs) such as erlotinib, gefitinib, and osimertinib showing significant clinical efficacy in patients harboring EGFR-mutant tumors. Similarly, chromosomal rearrangements involving the anaplastic lymphoma kinase (*ALK*) gene, most notably EML4-ALK fusions, result in aberrant activation of ALK signaling pathways. ALK-positive NSCLC patients have shown remarkable responses to ALK inhibitors like crizotinib, ceritinib, and alectinib, highlighting the success of precision medicine in oncology.^19^

In contrast, Kirsten rat sarcoma viral oncogene homologue (*KRAS*) mutations, particularly the KRAS G12C variant, are associated with smoking-related NSCLC and have historically been considered difficult to target due to the structure of the *KRAS* protein. However, recent breakthroughs have led to the development of *KRAS* G12C inhibitors such as sotorasib and adagrasib, offering new hope for patients with previously undruggable tumors. In addition to targeted therapies, identifying these mutations helps guide the use of immunotherapies, such as immune checkpoint inhibitors (e.g., pembrolizumab, nivolumab). While tumors with high PD-L1 expression and tumor mutational burden (TMB) may respond well to immunotherapy, the presence of certain driver mutations like *EGFR* or *ALK* can predict resistance, underscoring the need for mutation-specific treatment planning. Overall, integrating molecular profiling into clinical practice has transformed the therapeutic landscape of lung cancer, enabling personalized treatment approaches that improve patient outcomes and minimize unnecessary toxicity.^20^

### Colorectal cancer

Colorectal cancer (CRC) is one of the most common malignancies worldwide and a leading cause of cancer-related mortality. It often develops from precancerous polyps over many years, making it highly amenable to prevention through early detection. Screening plays a pivotal role in reducing both the incidence and mortality of CRC by identifying and removing these polyps before they progress to invasive cancer. Colonoscopy is considered the gold standard screening tool, as it allows direct visualization of the entire colon and rectum and enables immediate polyp removal during the procedure.^21^ Studies have shown that routine colonoscopy can significantly lower CRC-related death rates, particularly when initiated at the recommended age and intervals. In addition to colonoscopy, non-invasive stool-based tests, such as the fecal immunochemical test (FIT), are widely used for population-level screening. FIT detects hidden blood in the stool, a potential early sign of CRC, and is simple, cost-effective, and well-suited for annual screening. When used in combination with or organized screening programs, these tools greatly enhance the chances of diagnosing colorectal cancer at an early, more treatable stage, ultimately improving long-term patient outcomes and survival rates. As awareness and accessibility to screening improve, the burden of CRC is expected to decline significantly.^22^

### Skin cancer

Melanoma and nonmelanoma skin cancer (NMSC) are the two kinds of skin cancer. It is challenging to determine the exact prevalence of skin cancer due to underreporting and a lack of diagnostic criteria. Nonetheless, a number of epidemiologic investigations have revealed a rise in the frequency of NMSC and melanoma in recent decades. Malignant neoplasm diagnosis and treatment represent a significant health concern from the standpoint of patient welfare and medical expenses. Skin cancers frequently develop in sun-exposed areas of the head and neck, which can lead to major issues with diagnosis and treatment. Treatment options include immunotherapy, cryotherapy, radiation, chemotherapy, and surgical excision. Sunscreen use and appropriate sun protection measures are essential for preventing skin cancer.

## Importance of marine organisms in cancer drug discovery

Over 70% of the Earth's surface is covered by oceans, which serve as major habitats and harbor species with diverse biological and chemical characteristics, as well as acting as major habitats. Various drugs, drug candidates, and other metabolites from marine species have been found in recent years, despite the fact that most prescriptions are still issued on land. Since 2008, the number of known components has surpassed 30,000, with over 1000 new marine-derived chemicals being found yearly. They are frequently characterized as diversified, intricate, and architecturally unique. Until 2002, Faulkner annually reviewed new marine natural products in the *Natural Products Report*. Notably, Tyrian purple is the oldest known marine product, extracted from marine mollusks by the Phoenicians around 1600 BC. For many years, the marine natural product industry focused on the metabolites of marine algae and fish. Examples include polyunsaturated fatty acids like eicosapentaenoic acid and docosahexaenoic acid, marine biopolymers like agar and carrageenan, and the vitamins A and D found in fish liver oil. The actual method of manufacturing marine medications began in the 1950s when the Caribbean sponge Tethya crypta produced spongothymidine and spongouridine.^7^

The fungus Acremonium chrysogenum produces cephalosporin C, which was discovered in Mediterranean seawater samples close to Sardinia in the 1940s, establishing the cephalosporin antibiotic family.

Wein Heimer and Spraggins discovered that the gorgonian *Plexaura homomalla* contained a high concentration of prostaglandins in a 1969 investigation. A survey of maritime products from nature requests for patents showed a significant rise in the mid-1980s. The majority of the chemicals originate from marine invertebrates. Meanwhile, marine bacteria are becoming increasingly popular.

Biopolymers (polysaccharides: agar, carrageenan, alginates, antiviral sulfated polysaccharides via algae; chitin from crabs and its derivative chitosan); adhesive proteins from mussels; vitamins (A and D in the oils of fish livers from *Gadus morrhua* and *Hippoglossus hippoglossus*); minerals (iodine); UV-protective maritime substances (mycosporine acids, carotenoids, photolyase); and other marine cosmetics derived from the coral *Pseudopterogorgia elisabethae*, etc., are not covered in this review. Although it focuses on marine-derived cosmetics and cosmeceuticals, it provides a comprehensive overview of marine-derived nutraceuticals.^23^

The ocean is home to a vast array of organisms because of the varied habitats that the various maritime zones provide. Since the beginning of time, people have taken advantage of the sea's vast natural resources, including using fish and algae preparations as food and medicine. The traditional example of a substance obtained from the sea that has been used for eons is fish oil. A subfield of pharmaceutical science called marine pharmacology is concerned with compounds with active pharmacological characteristics found in marine plant and animal species. New bioactive natural compounds with structural and chemical properties frequently lacking in natural goods found on land can be found in the marine environment. Additionally, marine creatures offer a wealth of nutraceuticals and promising treatments for a number of human illnesses. Microbes are the current focus of marine pharmacology. One example of this is the identification of novel drug candidates from marine microorganisms. With over 13,000 molecules characterized, 3000 of which have active activities, there is a lot of promise for finding new chemicals in the ocean. Secondary metabolites make up the vast majority of marine natural compounds. Biological or conventional metabolic processes do not produce them and serve no significant role in a species' growth, development, or reproduction. Sixty-three percent of the new medications are classed as natural sources. For example, a synthetic molecule can act as a pharmacophore for a natural substance, whether it is modified, unaltered, or natural. Between 1981 and 2008, natural sources accounted for approximately 68% of all medications used to treat infections (including antibacterial, antiviral, antiparasitic, and antifungal substances) and 63% of cancer treatments.^24^

A significant portion of these drugs—11 out of 16—are used to treat different types of cancer in people. Furthermore, a great variety of dietary supplements that have been shown to be beneficial to human health are synthesized from extracts and semi-purified components of marine food. Many were thought to have antioxidant, immunostimulatory, and cancer-preventive qualities^25^

Numerous papers unequivocally show the link between dietary practices and cancer risk, aside from the medications derived from marine sources that have been licensed by the US and European authorities.^26^ Therefore, eating more seafood was linked to a lower risk of developing various malignancies, particularly colorectal and gastric cancers. It's interesting to note that several Asian countries' traditional folk medicine extensively uses certain marine creatures to treat various illnesses, including cancer.^27^ A few of the bioactive compounds that cause the health benefits mentioned above have been identified and reported; others are still being looked into. Numerous studies have demonstrated the potential of marine polyphenols to prevent cancer. These compounds, often isolated from brown algae, have shown an impressive array of biological activities, such as antitumor and antioxidant capabilities.^53^^,^^54^ The effect of brown algal polyphenols on ultraviolet B-induced skin carcinogenesis in the Skh-1 Hairless Mouse Strain (SKH-1) has been attributed to a reduction of pro-inflammatory *PTGS2* (cyclooxygenase-2, COX-2). ^29^

Phlorotannin dieckol's chemopreventive qualities were recently shown by Aicher et al. in an extensive *in vivo* investigation.^29^ This maritime polyphenol has been tested in an *in vivo* 7,12-Dimethylbenz[a]anthracene (DMBA) -induced skin cancer model. It was distinguishable in contrast to the brown algae Ecklonia cava, a Lessoniaceae family member. A daily diet might thereby considerably lower the incidence, magnitude, and burden of cancers caused by DMBA. Its protective mechanisms has been determined to involve (i) modulation of TNF-alpha, (ii) inhibition of NF-κB signaling, and (iii) antioxidant activity. Inhibiting IL-6 and IL-1β helps reduce inflammation. The latter was achieved by boosting glutathione levels while suppressing phase-I detoxification enzymes like cytochromes p450 and b5, and stimulating antioxidant enzymes (SOD, CAT, and GPx). Superoxide Dismutase, Catalase, and Glutathione Peroxidase. Dieckol suppresses pro-apoptotic (tumor protein p53 [*p53*], Bcl-2-associated X Protein [*Bax*], caspase-3, and -9) and anti-apoptotic (*Bcl-2, COX-2*, and *TGF-β1*) genes in drug-treated cells. The antioxidant impact has been quite noticeable.

Recently, dieckol and similar compounds have been shown to have a significant hepatoprotective effect in a number of liver injury models. Dieckol's hepatoprotective properties have therefore been documented in an *in vivo* model caused by tetrachloromethane. Dieckol's anti-apoptotic action in hepatocytes and the stimulation of antioxidant enzymes were proposed as the mechanisms of action. In models of liver injury caused by chemical agents, dieckol and other marine polyphenols may also have cancer-preventive qualities as a result of the aforementioned impact. In fact, an *in vivo* chemo-preventive efficacy was found in 2017. Dieckol was studied in rats using the N-nitrosodiethylamine-induced hepatocarcinoma model by Anjum et al.^30^ Dieckol enhances N-nitrosodiethylamine decontamination by suppressing carcinogen-activated first-generation enzymes (cytochrome P450, CYB5A, CYB5R, and CYPOR) while increasing phase II enzyme activity, glutathione S-transferase, and quinone reductase (GST and QR), in accordance with the authors.^31^ This marine polyphenol activates the intrinsic pathway, causing malignant altered cells to die by decreasing matrix metalloproteinases-2/-9 (MMP-2/9) and vascular endothelial growth factor (VEGF) and synthesis at the mRNA and protein levels, respectively. It has also been demonstrated that COX-2 and NF-κB, two pro-inflammatory factors, are suppressed. The current study did not investigate these effects, but these findings led the authors to speculate that dieckol would lessen carcinogen-induced angiogenesis, cancer cell invasion, and inflammatory processes.

## Role of marine in cancer

In aquatic and terrestrial environments, bacteria, fungi, plants, and animals' cells and tissues contain natural products (NP), commonly called secondary or specialized metabolites. They are fundamental components of modern pharmacology and have been used therapeutically since antiquity.^32^ Even though they are not necessary for an organism's vegetative growth, reproduction, or development, they boost competition with other species and are essential for the stress response, predator defense, preventing overgrowth by fouling organisms, and protection, according to the original observation that gave rise to the term “secondary metabolite.” against UV light and invasion by bacteria, viruses, or fungi.^33^ NPs are thought to be crucial for overcoming stressful situations brought on by varying or shifting environmental elements, including temperature, humidity, salinity, light intensity, or mechanical injury.^34^ Because they modulate complex interspecies interactions, where the lines separating various interaction types are not always clear or well defined For instance, commensalism, mutualism, and parasitism are three types of symbiosis. NPs are essential to the survival of organisms^35^. Therefore, survival and environmental adaptability processes encourage the synthesis of extremely complex and diverse compounds that have a great deal of promise for use as medications, and that tiny molecules cannot match.

In marine environments, complex bioactive metabolites frequently have detrimental or discouraging effects in environments like coral reefs, which are distinguished by fierce competition for food and space. Research on electron transport, quorum sensing, and cross-talk in communities of microbes, such as biofilms, has significantly transformed the comprehension of collaborative as well as competitive connections, rather than competitive ones, which may guarantee better survival tactics, especially in hostile settings.^36^

Quorum sensing has been found to be a crucial mechanism of regulation for cellular development and/or the production of secondary metabolites in a variety of bacteria, most notably streptomycetes. Finally, combined transcriptome and metabolomics research emphasized the need to explore microbial species interactions to uncover new secondary metabolites with different biological properties.^37^

The term “terrestrial organic compounds” has influenced drug discovery in a number of ways, including the highly effective analgesic morphine, which was isolated from opium^38^ and acts as an agonist of mu-opioid the receptors, inhibiting preliminary and postsynaptic processes in the ascending pain dissemination system^39^; the cardiotonic digitoxin, a secondary glycoside extracted from *Digitalis purpurea*,^40^ whose reduction of the Na^+^/K^+^ ATPase membrane pump raises intracellular sodium and calcium concentrations and encourages the encouragement of heart protein molecules with flexibility^41^; and salicylic acid from the bark of the willow tree, which was used as a pain reliever by the ancient Egyptians and Greeks^42^ and was the precursor to aspirin, the most common nonsteroidal anti-inflammatory drug (NSAID).

Penicillin is made from green. One of the most commonly used antibiotics, *Penicillium notatum*, was discovered in 1928.^43^ It binds to the bacterial DD-transpeptidase enzyme. There are four beta-lactam members that inhibit the synthesis of peptidoglycan cell walls. The taxane paclitaxel, which was originally derived from the outer layer of growth of the Pacific Coast yew tree *Taxus brevifolia*, and the vinca alkaloids (vincristine and vinblastine), which came from the Madagascar periwinkle plant *Catharanthus roseus*, are more recent examples of natural products that have impacted medicine, especially oncology. Upsetting the dynamic equilibrium of the microtubule polymerization or depolymerization jeopardizes the mitotic spindle system and causes apoptosis after metaphase arrest. Camptothecin, derived from the *Camptotheca acuminata* tree,^44^ effectively combats a variety of cancers^45^ by inhibiting DNA topoisomerase I.

Currently, almost 50% of all pharmaceuticals used in clinical settings are of natural origin, with anticancer medications having a larger percentage.

### Mechanisms of action of marine-derived compounds in cancer therapy

Marine-derived compounds exert their anticancer effects through a multitude of molecular mechanisms, including the induction of apoptosis, inhibition of angiogenesis, modulation of immune responses, and interference with key oncogenic signaling pathways. For instance, Didemnin B, isolated from the tunicate Trididemnum solidum, induces apoptosis by activating caspase cascades and inhibiting protein synthesis, leading to cell cycle arrest. Bryostatin-1, from *Bugula neritina*, modulates protein kinase C (PKC) activity, influencing both apoptotic and immune pathways, and enhancing the sensitivity of tumor cells to chemotherapeutic agents.^84^ In terms of angiogenesis inhibition, Trabectedin (ET-743) targets the tumor microenvironment by suppressing the transcription of genes involved in angiogenesis, such as *VEGF* and *IL-6*, and disrupts the DNA binding of transcription factors in the NF-κB pathway. Squalamine, derived from the dogfish *Squalus acanthias*, is a cationic steroid that inhibits angiogenesis by blocking Na^+^/H^+^ exchange and VEGF-mediated endothelial cell proliferation.^46^

Regarding immune modulation, Marizomib (Salinosporamide A), a marine-derived proteasome inhibitor, disrupts NF-κB signaling, which is critical in inflammation and tumor immune evasion. It enhances tumor cell recognition by cytotoxic T cells and reduces the expression of immune checkpoint proteins. Largazole, from marine cyanobacteria, is a histone deacetylase (HDAC) inhibitor that epigenetically reprograms tumor cells and upregulates tumor-suppressor genes, increasing immune-mediated cytotoxicity. Marine-derived compounds also interact with several critical cancer signaling pathways. For example, Halichondrin B and its analog Eribulin inhibit microtubule dynamics, indirectly affecting the PI3K/AKT/mTOR pathway and promoting apoptosis. Fucoidan, a sulfated polysaccharide from brown algae, has been shown to inhibit the Wnt/β-catenin pathway, reducing proliferation and inducing differentiation in colon cancer cells. Moreover, Lamellarin D, from the marine mollusk Lamellaria sp., induces mitochondrial-mediated apoptosis and inhibits topoisomerase I, with downstream effects on p53 activation.^47^

These mechanistic insights underscore the multi-targeted nature of marine-derived compounds and highlight their potential in combination therapies with conventional chemotherapeutics or targeted agents. Understanding these pathway-specific interactions is crucial for optimizing the clinical application of these promising natural products.

### How are marine drugs better than conventional chemotherapeutic agents in treating cancer?

Natural products have significantly contributed to cancer therapy development, as they account for over 70% of clinically used compounds.^87^^,^^88^ Plant-derived antineoplastic analogues commonly used in medicine include podophyllotoxin and its analogues etoposide (Etopophos®), teniposide (Vumon®), and Vinblastine (Velban®), vincristine (Oncovin®), and their respective analogs vindesine (Eldisine®), vinorelbine, and belotecan (Camptobell®); paclitaxel (Taxol®) and its derivatives, such as topotecan (Hycamtin®) and irinotecan (Camptosar®). As evidenced by the anthracyclines doxorubicin (Doxil®; Adriamycin®), daunorubicin (Cerubidine®), and epirubicin (Ellence®), the glycopeptide bleomycin (Blenoxane®), and the non-ribosomal peptide dactinomycin (Cosmegen®), soil microorganisms represent a significant source of chemotherapeutic medicines.^48^

Biological systems undergo phenotypic alterations as a result of the structural requirements found in the unique and compact configuration of natural molecules that facilitate molecular interactions or binding to specific targets. These characteristics are directly linked to their physiological ability and effectiveness as therapies in a wide spectrum of diseases. According to estimates, natural products were employed to generate 64% of all currently licensed drugs.^49^ Because of their priceless biological diversity, there is a remarkable chance that new naturally occurring anticancer medications may be discovered. Less than 2% of plants are thought to have had their antineoplastic ingredients examined yet; even in those cases, only cytotoxic action was looked for. Actually, many recognized compounds have previously unheard–of properties when evaluated against novel therapeutic targets, and many compounds with antineoplastic properties derived from marine animals, microbes, and vegetation are still to be discovered.^50^ Rarely have maritime plants been addressed as a separate and independent category in the literature. Because of their taxonomic and morphological resemblance to freshwater algae, marine plants' fundamental “marine-ness” tends to diminish. In marine biology textbooks and courses, these plants are typically regarded as either the inferior relatives of marine animals or as representative examples of specific groups of algae. Algae make up more than 90% of marine plant species. Due to maritime plants, such as mangroves and marine algae, have a wide variety of chemicals. Goods that are separated from these plants have demonstrated cytotoxic, analgesic, anti-inflammatory, antibacterial, antifungal, hypotensive, and spasmogenic properties.

Oceans cover more than 70% of the globe. An estimated 5 × 10^8^ bacterial and eukaryotic species comprise the world's biodiversity. With around 250,000 known species, the marine environment is, in fact, a remarkably varied source of life.^51^ A total of 3.7 × 10^30^ microorganisms have been found in marine habitats,^52^ and even though 99% of bacteria cannot be grown, they can still produce a variety of interesting natural chemicals that may be exploited as potential treatments.^53^ Marine species' remarkable chemical and pharmacological diversity may be explained by their need to create secondary metabolites as defensive mechanisms to withstand pressure, salinity, and temperature extremes, as well as to fend off predators. In Europe, China, India, and the Near East, sea vegetation has been utilized for healing reasons since ancient times.^54^

Less than 5% of the deep ocean floor has been explored, and little over 0.01% has been thoroughly sampled.^55^ The Caribbean sponge (*Cryptotethya crypta*) was the first living aquatic creature to be chemically analyzed,^56^ before cytosine arabinoside (ara-C) was discovered.^56^ Between 1950 and 1960, various botanical researches were conducted on pure chemicals produced from this bacterium. Furthermore, various marine organisms—recognized as prolific sources of bioactive metabolites for anticancer drug discovery—have been extensively studied, encompassing invertebrates such as sponges, soft corals, sea fans, sea hares, nudibranchs, bryozoans, and tunicates, alongside microbes (i.e., bacteria, actinobacteria, cyanobacteria, and fungi), microalgae, and macroalgae (i.e., seaweeds).^57^ A number of animals have been studied for their potential to prevent cancer Additionally, as marine chemistry continues to advance, new methods like metabolomics have been employed to study marine compounds from different perspectives.^58^

**Figure 3:**
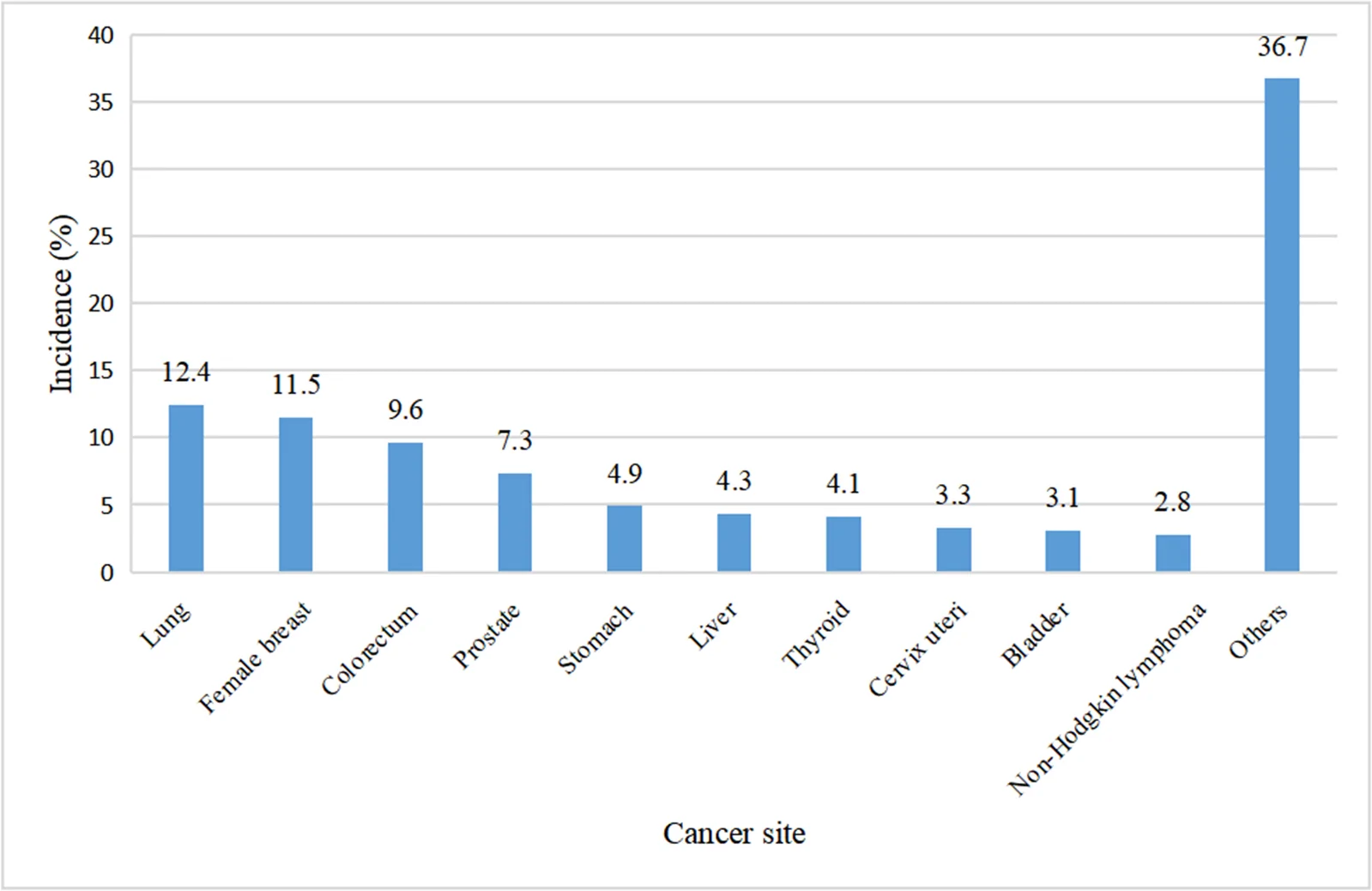
Global cancer incidence based on GLOBOCAN 2022 data. Other cancers accounts for 36.7% (rare or unspecified cancers), followed by lung cancer at 12.4%, female breast cancer at 11.5%, colorectal cancer at 9.6%, prostate cancer at 7.3%, stomach cancer at 4.9%, liver cancer at 4.3%, thyroid cancer at 4.1%, and cervical cancer at 3.3%.

Approximately 12.4% and 11.5% of cancer cases in recent years have been lung cancer and breast cancer, 9.6% have been colorectal cancer, and 7.3%, 4.9%, 4.3%, 4.1% and 3.3% have been prostate, stomach, liver, thyroid and cervical cancer, respectively [Figure 3].^59^ However, if we pay great attention, we can find that more than 36.7% of cancer diagnoses worldwide in recent years are either rare cancer diseases or some other kind of cancer that the general public is unaware of.

**Figure 4:**
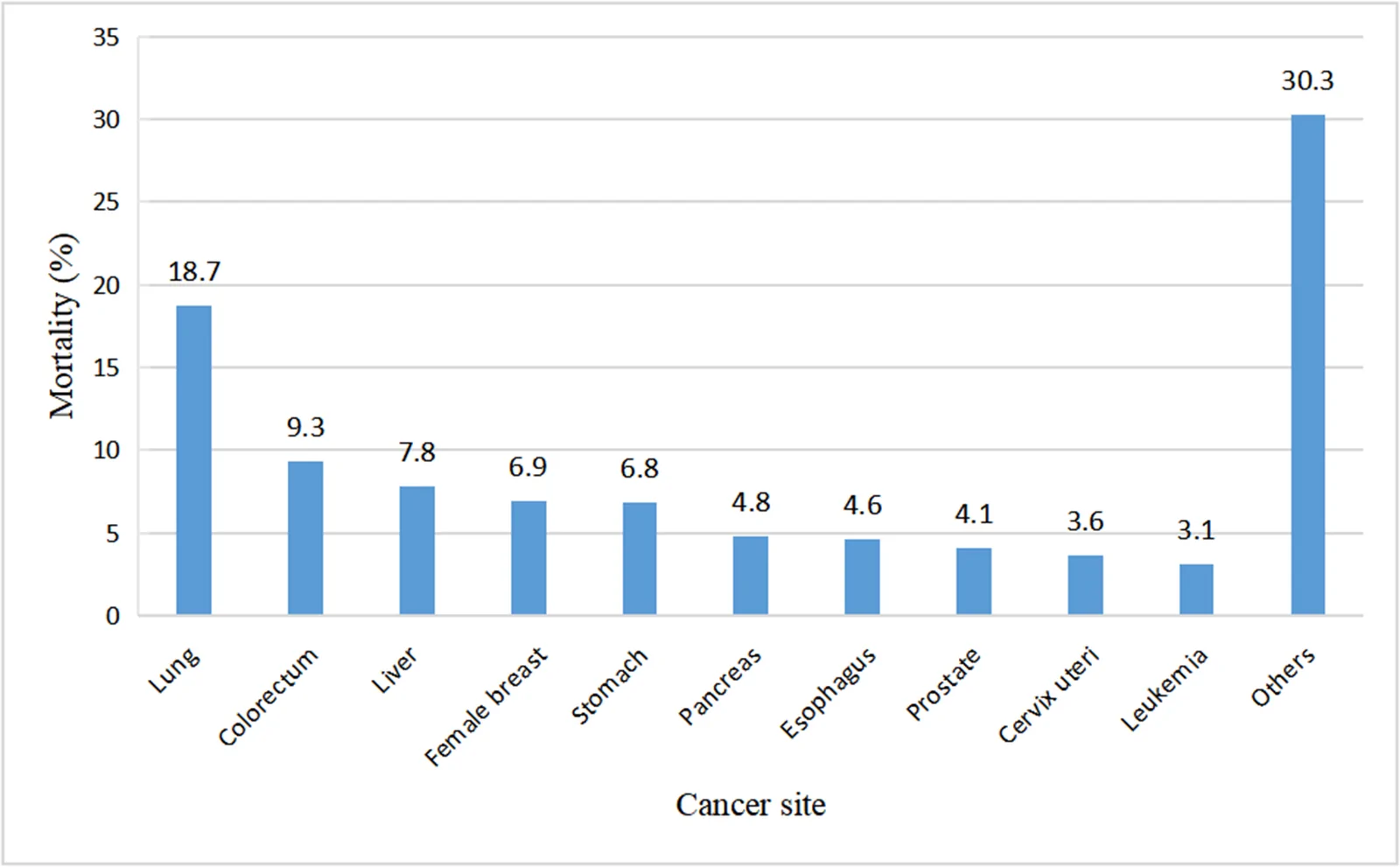
Global cancer mortality rates by cancer site (2022, GLOBOCAN data). Lung cancer as a cancer type has the highest number of deaths (18.7%), followed by colorectal cancer (9.3%), liver cancer (7.8%), breast cancer (6.9%), stomach cancer (6.8%) pancreatic cancer (4.8%), esophageal cancer (4.6%), prostate cancer (4.1%), and other cancers (30.3%).

We can understand that in recent times, about 18.7%, 9.3% and 7.8% of deaths cases are caused by lunge cancer, colorectal cancer and liver cancer, 6.9% and 6.8% deaths are caused by breast cancer and stomach cancer, less than 5.0% pancreatic cancer, esophageal cancer and prostate cancer, as we observe the chart, we can see that more 30.3% of deaths caused by cancer in recent times in the world are either rare cancer diseases or some other type of cancer of which the current world doesn't have any idea about [Figure 4].^60^

**Figure 5:**
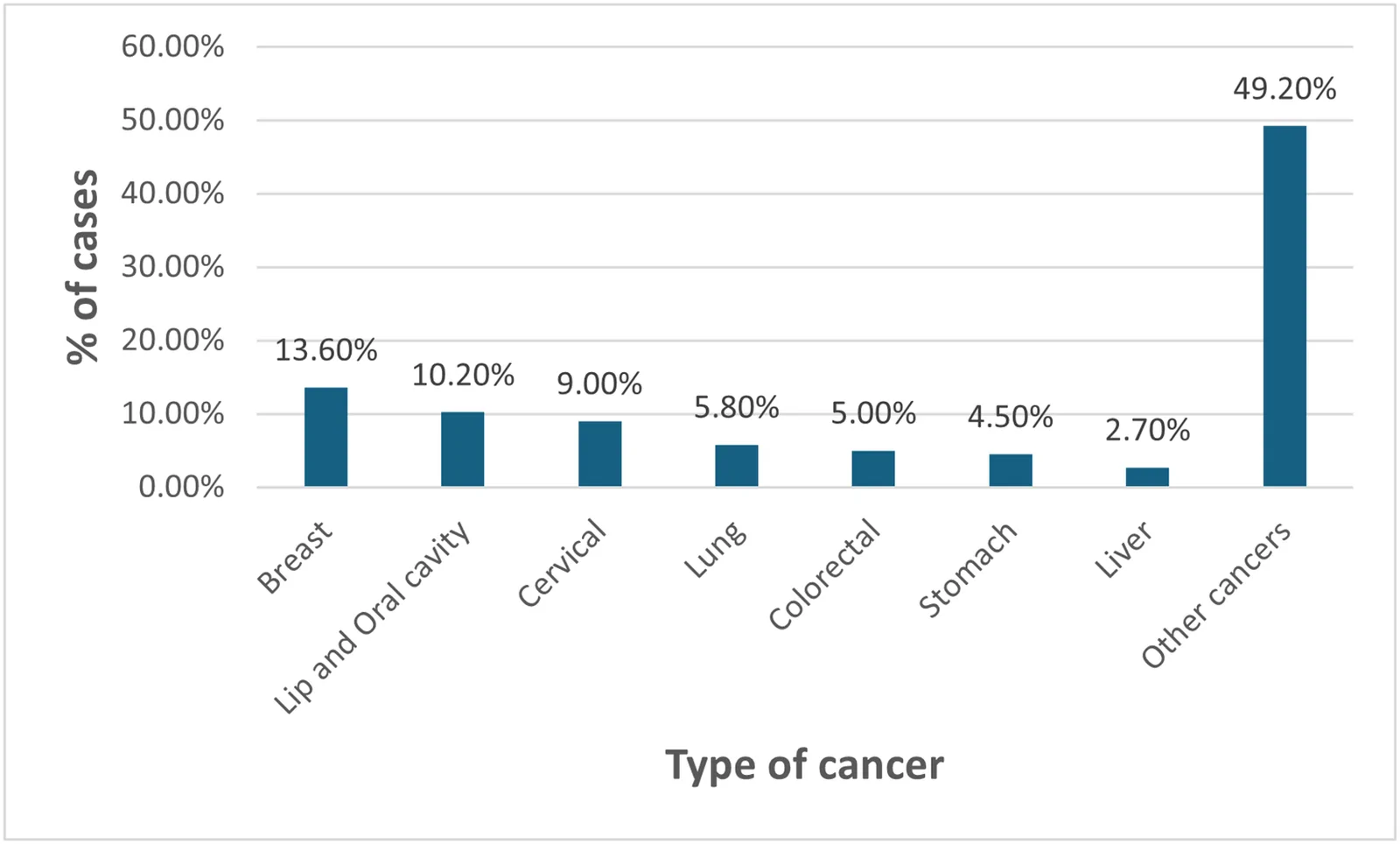
Distribution of cancer incidence in India by type (GLOBOCAN 2022). Breast cancer is the most common cancer in India (13.6%), followed by lip and oral cavity (10.2%) and cervical cancer (9.0%). Lung (5.8%), colorectal (5.0%), stomach (4.5%), and liver cancers (2.7%) are also significant contributors. Other cancers collectively account for 49.2% of cases.

In India, breast cancer represents the highest proportion of cancer cases (13.6%), followed by cancers of the lip and oral cavity (10.2%) and cervical cancer (9.0%). Lung cancer accounts for 5.8% of cases, while colorectal, stomach, and liver cancers contribute 5.0%, 4.5%, and 2.7%, respectively. Other cancer types collectively comprise 49.2% of the total cancer burden in India [Figure 5].^59^, ^60^, ^61^

## Classification of various marine-derived active ingredients

### Microalgae

**Figure 6:**
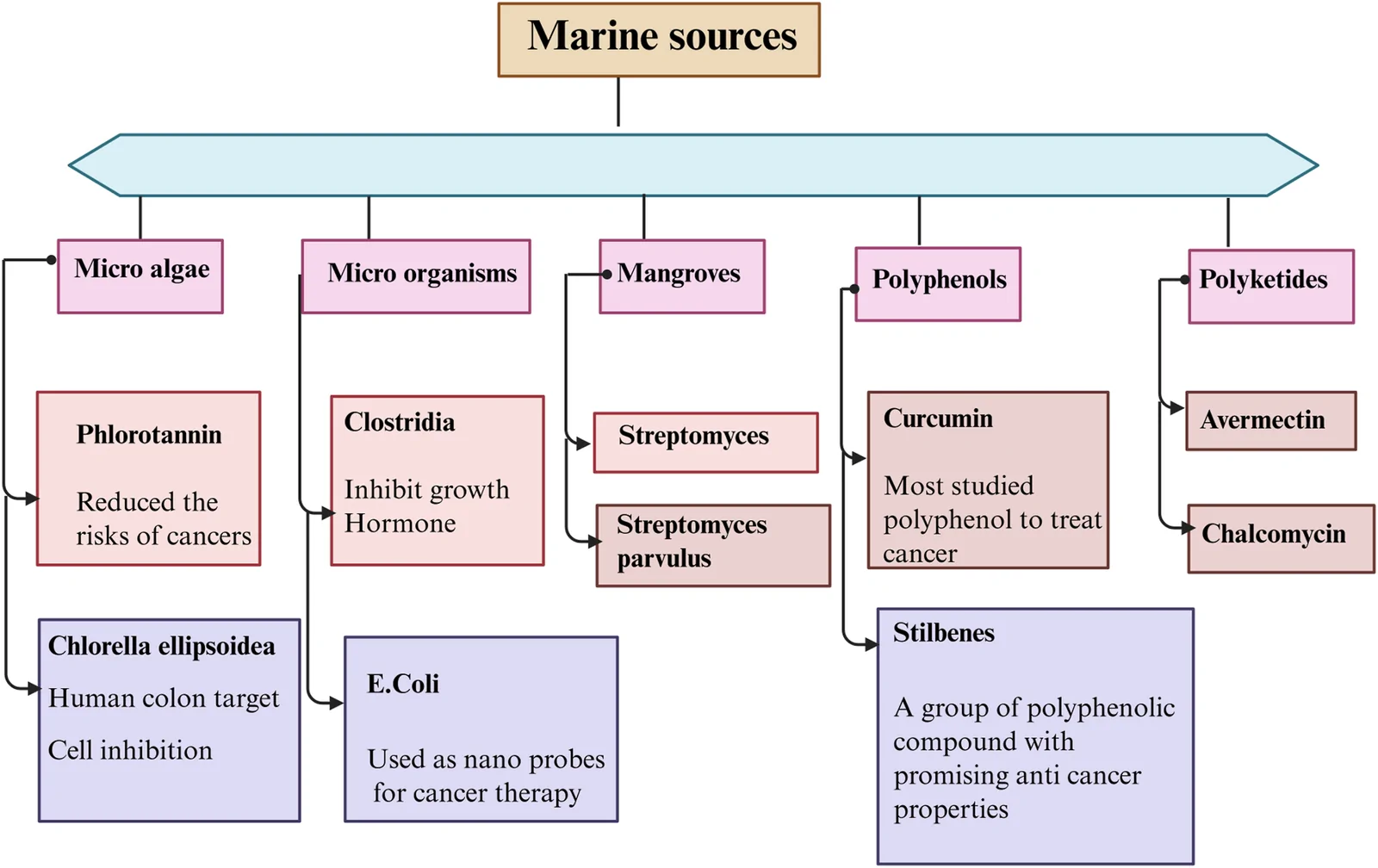
Sources and corresponding bioactive compounds of marine drugs. *E. coli*: *Escherichia coli*.

Up to 40% of the world's production may come from eukaryotic plants known as microalgae. They have short generation times (some species double within 5–8 h), are located at the base of aquatic food webs, and have expanded to practically every biotope, from temperate to extreme (for example, hydrothermal vents and cold conditions). Their metabolic versatility, which allows them to commence the synthesis of many molecules with potential uses across numerous biotechnology areas (e.g., food, energy, health, the surroundings, and biomaterials), provides an edge in marine drug development.^62^ They are a renewable and underused resource for drug research that can be easily produced in photo-bioreactors (e.g., 100,000 L bioreactors) to generate a large biomass They eliminate the elements nitrogen and phosphorus instruments such as derivatives which may be contaminants contingent upon their content, and fix CO_2_ and use solar energy, which assists in minimizing the impacts of the release of greenhouse gases [Figure 6 & Table 1].^63^

**Table 1:** Marine microalgae-derived compounds and their anticancer activity against human cancer cell lines.

Microalgae	Compound/Fraction	Cells of Interest	Reference
Skeletonema costatum, Pseudonitzschia delicatissima, and Thalassiosira rotula.	Aldehydes that are PUA	Colon adenocarcinoma (Caco-2) Lung adenocarcinoma (A549) Adenocarcinoma of the colon (COLO 205)	64
*Chlorella ellipsoidea*	Carotenoid extract	The HCT-116 colon cancer	65
*Acus Synedra*	The polysaccharide chrysolaminaran	Colorectal adenocarcinoma (HT-29 and DLD-1)	66
*Dunaliella tertiolecta*	It has previously been discovered that C. ellipsoidea contains the pigment violaxanthin.	Breast adenocarcinoma MCF-7	67
*Cocconeis scutellum*	Eicosapentaenoic acid, or EPA,	Breast cancer (BT20)	68
*Phaeodactylum tricornutum, Odontella aurita, Chaetoseros* sp.*, and Cylinrotheca closterium*	A carotenoid called fucoxanthin	Prostate cancer includes three types (PC-3, DU145, and LNCaP), colon adenocarcinoma (HT-29), promyelocytic leukemia (HL-60), DLD-1, and Caco-2	69
*Calcitrans Chaetoceros*	An excerpt of the EtOH extract from AcOEt	Breast cancer of MCF-7 (MDA-MB-231)	67
The Amphidinium carterae	Proportion of hexane's CH3Cl component The percentage of AcOEt	Melanoma of the Skin (B16F10), A549, HL-60 HL60	70
Eleven benthic diatom strains, such as Amphidinium operculatum and Ostreopsis ovata	Extract of MeOH	HL-60	71
*Navicula incerta*	Stigmasterol, also known as phytosterol.	Liver hepatocellular carcinoma (HepG2)	72
*Phaeodactylum tricornutum.*	Glycerols monogalactosyl 1 and NAMO, commonly referred to as fatty alcohol ester.	Epithelial cell lines of HL-60 mice (W2, D3)	73
The skeletal muscle of the costatum The skeleton of the marinoid	Hydrophobic Fraction + Hydrophobic Fraction for PUAs	Caco-2 (A2058-unaffected) The cutaneous melanoma (A2058)	74
Canada's marine microalgal pool	Aquatic extract	MCF-7, osteosarcoma (MNNG), pancreatic adenocarcinoma (BxPC-3), stomach cancer (N87), lung cancer (H460), prostate cancer (PC-3, DU145),	75

A2058: Cutaneous melanoma cell line; A549: Human lung adenocarcinoma cell line; AcOEt: Ethyl acetate (solvent used in extraction); BxPC-3: Pancreatic adenocarcinoma cell line; BT20: Human breast cancer cell line; Caco-2: Human colorectal adenocarcinoma cell line; CH_3_Cl: Methyl chloride (solvent); COLO 205: Colon adenocarcinoma cell line; DLD-1: Human colon adenocarcinoma cell line; DU145: Prostate cancer cell line; EPA: Eicosapentaenoic acid; EtOH: Ethanol (solvent); Fucoxanthin: A carotenoid pigment found in brown algae; H460: Human lung cancer cell line; HCT-116:Human colon cancer cell line; HepG2: Human liver hepatocellular carcinoma cell line; HL-60: Human promyelocytic leukemia cell line; HT-29: Human colorectal adenocarcinoma cell line; LNCaP: Human prostate cancer cell line; MCF-7: Human breast adenocarcinoma cell line; MDA-MB-231:Human breast cancer cell line; MNNG: Human osteosarcoma cell line; NAMO: Nonyl-8-acetoxy-6-methyloctanoate; N87: Human stomach cancer cell line; PC-3: Human prostate cancer cell line; PUA: Polyunsaturated aldehydes; W2, D3: Mouse epithelial cell lines.

### Mangrove

Because of the variety of ecosystem services they offer, including improving fisheries, storing and sequestering carbon, and protecting coastlines from the many effects of natural disasters, mangroves rank among the world's most economically and ecologically significant ecosystems. Additionally, neutralizing aquatic and terrestrial contaminants and cycling nutrients enhances the quality of coastal and nearshore waters and safeguards the marine ecosystem. By giving them a home and food, they also sustain wildlife (mammals, reptiles, birds, etc.) and marine life (fish, shrimp, crabs, etc.) species.^76^ Anaerobic soil, high salinity, high temperatures, strong winds, and other extreme environmental conditions are all characteristics that *A. marina*, a type of mangrove, can withstand well. Overly saline water can stunt the growth of the grey mangrove, but in fresh and saltwater waterways, they flourish and grow to their full height. The species can withstand high salinity by excreting salts through its leaves [Table 2].^77^^,^^78^ Researchers investigated at the plant's potential for medicinal applications and drug discovery, driven by its remarkable resilience.

**Table 2:** Sources of Mangrove-Derived compounds and their cytotoxic effects on diverse cancer Cell lines.

Strain	Source	Compound/Crude Extract	Reference
211726 Streptomyces sp.	Heritiera globosa mangrove forests rhizosphere soil (China)	Azalomycin F4a 2-ethylpentyl ester and Azalomycin F5a 2-ethylpentyl ester are the first two.	79
FMA Streptomyces sp.	Chinese mangrove soil	Streptocarbazole A and B,	80
CBJ1 Streptomyces parvulus	The Indian mangrove soil	D-actinomycin	61
H74-21 strain of Streptomyces antibioticus	Sediment from a Chinese mangrove area	C-Streptomyceamide	81
Cheonanensis Streptomyces VUK-A	Silt from the Indian Coringa's mangrove ecosystem	Phthalate of methyl butyl propyl	82
219807 Streptomyces sp.	Chinese mangrove soil	In addition to 2-methyl-11,11′-O-dimethylelaiophylin, halicoblelide D ∗ 11-O-methyl-elaiophylin (5), 11,11′-O-dimethylelaiophylin (6), efomycin G (7), and elaiophylin (4)	83
ACT01, ACT02, ACT03, ACT04, and ACT05 are Streptomyces species.	The Manakkudi (India) environment's mangrove sediment	Extract of crude ethyl acetate	84
Streptomyces pluripotens MUSC 137T	The Malaysian mangrove soils	When methanol is extracted crudely	85
MSU3 Streptomyces olivaceus	*Rhizophora mucronata*, or mangrove rhizosphere soil, is found in India.	Crude ethyl acetate extract	86
Streptomyces sp. MUM256.	The Malaysian mangrove soils	Ethyl acetate extract	87

ACT01–ACT05: *Streptomyces* strains isolated from Manakkudi mangrove sediment (India); CBJ1: *Streptomyces parvulus* strain from Indian mangrove soil; C-Streptomyceamide: A compound isolated from *Streptomyces* antibioticus H74-21; FMA: *Streptomyces* sp. strain from Chinese mangrove soil; H74-21: Strain of *Streptomyces antibioticus* from Chinese mangrove sediment; MUM256: *Streptomyces* sp. strain from Malaysian mangrove soils; MSU3: *Streptomyces olivaceus* strain from Indian mangrove rhizosphere; MUSC 137T: *Streptomyces pluripotens* strain from Malaysian mangrove soils; VUK-A: *Streptomyces cheonanensis* strain from Indian Coringa mangrove silt.

### Marine organisms

Marine microbes, particularly actinobacteria and cyanobacteria, have emerged as prolific producers of structurally diverse and pharmacologically potent anticancer agents. These microorganisms thrive in extreme marine environments, which drive the evolution of unique metabolic pathways and secondary metabolites not commonly found in terrestrial organisms.^150^ Among actinobacteria, the genus Salinispora has gained notable attention, especially with the discovery of Salinosporamide A (Marizomib)—a potent proteasome inhibitor derived from Salinispora tropica. Marizomib exhibits broad-spectrum antitumor activity and is currently undergoing clinical trials for treating multiple myeloma and glioblastoma, showcasing its potential in targeting proteasome-mediated cancer cell survival. Similarly, Lomaiviticins A and B, complex polyketide antibiotics isolated from marine actinobacteria (Micromonospora species), possess remarkable cytotoxic activity through DNA double-strand break induction, making them promising leads in cancer chemotherapy.^88^

Cyanobacteria also contribute significantly to the marine pharmacopeia, producing compounds such as curacin A, cryptophycin, and dolastatin derivatives, which interfere with microtubule dynamics or act as DNA-damaging agents. These microbial-derived molecules often serve as either direct drug candidates or scaffolds for semi-synthetic modification to improve pharmacological profiles [Table 3]. The ability to culture these microorganisms, sequence their biosynthetic gene clusters, and manipulate their metabolic pathways through genetic engineering and synthetic biology further enhances their value in drug discovery. As research advances, marine microbial metabolites are expected to play an increasingly central role in the development of next-generation anticancer therapies.^89^^,^^90^

**Table 3:** Marine-Origin compounds with reported anticancer activity.

Marine source	Component	Targeted cancer or cell lines	Reference
*Ecteinascidia turbinata*	Ecteinascidin/Trabectedin (alkaloid)	MFC7 A549	91
Dolabella auricularia/Symploca sp. VP642	Brentuximab and Vedotin (antibody–drug conjugate)	The cells of non-Hodgkin's lymphoma (Karpas 299)	92
The cryptotheca crypta	Nucleoside cytarabine	Cells from Acute Myeloid Leukemia (AML)	93
CNC139, Aspergillus sp.	(Diketopiperazine) Plinabulin	MM.1S, MM.1R, RPMI8226, and INA-6 myeloma cells	94
*Aplidium albicans*	Plitidepsin, also known as depsipeptide.	MCF-7	95
*Halichondria Okadai*	Lurbinectedin is an alkaloid.	Ovarian cancer cells include RMG1, RMG2, KOC7C, HAC2, A2780, HeyA8, and SKOV-3.	96

A2780: Human ovarian cancer cell line; A549: Human lung adenocarcinoma cell line; AML: Acute Myeloid Leukaemia; CNC139: Marine-derived fungal strain (Aspergillus sp.); HeyA8: Human ovarian cancer cell line; HAC2:Ovarian cancer cell line; INA-6: Human multiple myeloma cell line; KOC7C: Human ovarian cancer cell line; Karpas 299: Non-Hodgkin's lymphoma cell line; MCF-7: Human breast adenocarcinoma cell line; MM.1R: Multiple myeloma cell line, resistant; MM.1S: Multiple myeloma cell line, sensitive; RPMI8226:Human multiple myeloma cell line; RMG1:Human ovarian cancer cell line; RMG2: Human ovarian cancer cell line; SKOV-3:Human ovarian adenocarcinoma cell line.

### Polyphenols

A family of natural chemicals generated from plants, polyphenolic substances have some hydroxyl groups and at least one aromatic ring. They are extensively found in everyday dietary sources and are mostly derived from secondary metabolites of plants.^97^^,^^98^ Numerous subtypes of polyphenolic chemicals exist, such as flavones, tannins, phenolic acids, anthocyanins, and others.^99^^,^^100^ Many traditional doctors in ancient China used herbal remedies like S. glabra (also known as Zhong Jie Feng) and *Xanthoceras sorbifolium* Bunge to treat rheumatism, abscesses, and other ailments. Polyphenols were later found to be the active ingredients in these remedies [Table 4].^101^^,^^102^ Recent pharmacological researches have shown that polyphenolic substances have clear anticancer potential and substantial antioxidant benefits.^103^^,^^104^ Polyphenolic compounds formed from natural products have sparked a lot of interest in the battle against cancer due to their abundance of resources and low toxicity.^105^^,^^106^ Polyphenolic compounds made from natural sources do, however, have several limitations that restrict their use in the clinical treatment of malignancies. These limitations include low stability, poor targeting ability, and poor solubility and bioavailability.^107^^,^^108^

**Table 4:** Effect of polyphenols on different cancer cell lines.

Cancer	Different cell types	The principal repercussions	Reference
Quercetin-induced hepatocellular cancer	HepG2 (SMMC-7721)	Causing apoptosis to reduce the growth of tumors	109
Cancer of the Breast	MCF-7	Stopping the process of apoptosis	110
Colon cancer	HCT-116	Induction of Apoptosis	111
Gastric cancer	BGC-823	Induction of Apoptosis	112
Primary effusion lymphoma-related cancer	BC1, BCBL1, and BC3	PI3K/AKT/mTOR pathway repression, autophagy induction, and apoptosis	113
Cervical cancer	HeLa	Causing apoptosis and autophagy	114
Hepatocellular carcinoma	LM3	Causing autophagy, apoptosis, G2/M cell cycle arrest, and JAK/STAT3 pathway inhibition.	115
Cancer of the pancreas	PATU-8988 and PANC-1	Blocking the STAT3 pathway and metastasis	116
Cancer of the prostate	PC-3	Halting angiogenesis and tumor development	117
Retinoblastoma	Y79	Inhibits migration, angiogenesis, and apoptosis.	118
Retinal and rhesus choroidal cancer	RF/6A	Preventing angiogenesis and triggering apoptosis	119
Leukemia	Molt-4, HSB2, CEM, and Jurkat	PI3K/AKT pathway blockade and apoptosis activation	120
Lung cancer	A549	Apoptosis induction and PI3K/AKT pathway inhibition	121
Ovarian cancer	SK-OV-3, A2780	Triggering autophagy and apoptosis while blocking the AKT/mTOR/p70S6K pathway	122
Oral cancer	YD-38, SCC-VII, and SCC-25	Leading to death and G2/M cell cycle halt	123
Tongue cancer	The SCC-4	Stop metastases and the *ERK pathway*.	124
Cholangiocarcinoma	HCCC9810, QBC939	Induction of apoptosis, suppression of PI3K/AKT, and reduction of tumor growth	125

AKT: Protein Kinase B; BC1, BCBL1, BC3: Primary effusion lymphoma cell lines; BGC-823: Human gastric cancer cell line; CEM: Human T-cell acute lymphoblastic leukaemia cell line; ERK: Extracellular signal-regulated kinase; G2/M: Gap 2/Mitosis cell cycle checkpoint; HeLa: Human cervical cancer cell line; HCCC9810: Human cholangiocarcinoma cell line; HCT-116: Human colon cancer cell line; HSB2: Human acute lymphoblastic leukaemia cell line; JAK/STAT3: Janus kinase/signal transducer and activator of transcription 3 pathway; Jurkat: Human T-cell leukaemia cell line; LM3: Hepatocellular carcinoma cell line; MCF-7: Human breast adenocarcinoma cell line; Molt-4: Human leukaemia cell line; PATU-8988: Pancreatic cancer cell line; PANC-1: Human pancreatic carcinoma cell line; PC-3: Human prostate cancer cell line; PI3K: Phosphoinositide 3-kinase; QBC939: Cholangiocarcinoma cell line; RF/6A: Rhesus monkey retinal choroidal endothelial cell line; SCC-25, SCC-VII, SCC-4: Oral and tongue squamous carcinoma cell lines; SMMC-7721: Human hepatocellular carcinoma cell line; SK-OV-3: Human ovarian adenocarcinoma cell line; STAT3: Signal transducer and activator of transcription 3; Y79: Human retinoblastoma cell line; YD-38: Human oral cancer cell line.

### Polyketide

The broad family of natural substances known as polyketide compounds contains a wide range of intricate structures. Many of these medications are prized for their powerful biological effects; lovastatin, rifamycin, tetracycline, and erythromycin constitute some of the most well-known instances. Polyketides are synthesized by polyketide synthases (PKSs), a class of enzymes that assemble acyl coenzyme A (CoA) building blocks in an assembly line-like manner.^126^ Type I and type II PKSs have three main catalytic domains: the acyltransferase (AT) domain, which at first chooses and loads the component parts; the acyl carrier protein (ACP) domain, which grows longer the polyketide chain; and the ketosynthase (KS) domain, which catalyzes decarboxylative Claisen condensations for chain extension [Table 5]. There may be additional enzymes. The diversity and complexity of polyketides that may be generated are considerably boosted by these enzymes, which can change the growing polyketide chain during or after assembly, either alone or in cooperation with the PKS mega synthase. There has long been interest in designing these enzymes to generate new polyketides in a predictable fashion because of the innate modularity of many PKSs and their huge potential for producing compounds with medical utility. From defining PKSs with particular properties to acquiring a fundamental knowledge of how these enzymes operate, to changing catalytic domains and modules to make synthetic products, decades of study have enhanced our understanding of this endeavor.^127^^,^^128^ We examined recent advancements in these disciplines in this section, emphasizing both the new and the newly gained understanding of PKSs and the tools produced to help PKS engineering projects. Although a vast amount of their jobs have been conducted regarding PKSs, here we've narrowed our talk to a few selected issues from current PKS research.

**Table 5:** Different types of Polyketides & their sources.

Polyketide	Structure	Producer	References
Avermectin	16-Membered macrolide ring	Streptomyces avermitilis streptomyces	129
Chalcomycin	16-Membered macrolide ring	Streptomyces bikiniensis and Streptomyces	129
Candicidin	Polyene macrolide with a 38-membered ring	IMRU 3570 Streptomyces griseus	130
Tacrolimus FK506	23-Membered macrolide ring	Streptomyces tsukubaensis and Streptomyces sp. MA6858	131
FK520 (Ascomycin)	23-Membered ring of macrolides	Ascomyceticus var. Hygroscopic Streptomyces	132
Tautomycin	Linear	CK4412 Streptomyces sp.	133
Tylosin	16-Membered ring of macrolides	Fadiae Streptomyces	134
The medication medermycin	Octataketide	K73 Streptomyces sp.	135
Doxorubicin	Decaketide	Little Streptomyces	136
Oxytetracycline	Decaketide	Rizomyces streptomyces	137
Hedamycin	Dodecaketide	Grizeoruber Streptomyces	138
Fredericamycin	Pentadecaketide	ATCC 49344 Streptomyces griseus	139

ATCC: American Type Culture Collection; CK4412: A strain of *Streptomyces* sp. producing Tautomycin; FK506 (Tacrolimus): 23-membered macrolide produced by *Streptomyces tsukubaensis*; FK520 (Ascomycin): 23-membered macrolide from *Streptomyces hygroscopicus* var. *ascomyceticus*; IMRU 3570: Strain of *Streptomyces griseus* producing Candicidin; K73: *Streptomyces* sp. strain producing Medermycin; MA6858: A strain of *Streptomyces* sp. that also produces Tacrolimus.

## Current status of clinical trials for marine-derived anticancer agents

Marine-derived compounds have steadily advanced through the drug development pipeline, with several progressing into clinical trials, highlighting their therapeutic promise. As of recent updates, over 20 marine-derived molecules are in various phases of clinical evaluation for cancer treatment. Notable examples include Plitidepsin (Aplidin), a cyclic depsipeptide from the tunicate Aplidium albicans, which has shown anti-myeloma activity and is currently in Phase III trials in combination therapies. Marizomib (Salinosporamide A), derived from the marine actinobacterium Salinispora tropica, is a potent proteasome inhibitor being evaluated in Phase I/II trials for glioblastoma and multiple myeloma. Largazole, a marine cyanobacterial-derived histone deacetylase (HDAC) inhibitor, is undergoing preclinical and early-phase clinical investigations for its efficacy against solid tumors. Other candidates such as Bryostatin-1, from the bryozoan Bugula neritina, are being studied in combination therapies for hematological malignancies and Alzheimer's disease, due to their ability to modulate protein kinase C. Ecteinascidin 743 (Trabectedin), derived from the tunicate Ecteinascidia turbinata, has already gained FDA approval for the treatment of soft tissue sarcoma and relapsed ovarian cancer. Eribulin mesylate, a synthetic analog of halichondrin B from the sponge Halichondria okadai, is approved for metastatic breast cancer and liposarcoma, and continues to be investigated in trials for additional indications.^140^

Furthermore, several marine-derived molecules are in Phase I trials, including Dolastatin 10 derivatives and Soblidotin, being evaluated for their microtubule-inhibiting properties in solid tumors. With advances in formulation science and biomarker-driven patient selection, many of these agents are now being evaluated in targeted therapy settings, particularly in tumors with defined genetic alterations. The increasing inclusion of marine natural products in precision medicine clinical trials underscores their growing importance in oncology [Table 6]. Regular updates from databases such as ClinicalTrials.gov reflect a dynamic and expanding portfolio, with over 100 marine-derived compounds in various preclinical and late-stage clinical testing stages, pointing toward a promising horizon for marine-based cancer therapeutics.^141^

**Table 6:** List of marine-derived anticancer drugs — approved vs. pipeline candidates.

Drug Name	Source Organism	Mechanism of Action	Cancer Type	Status	Ref
Trabectedin (Yondelis)	*Ecteinascidia turbinata* (tunicate)	DNA minor groove binding; inhibits transcription	Soft tissue sarcoma, ovarian	FDA Approved	142
Eribulin (Halaven)	Synthetic analog of halichondrin B (*Halichondria okadai*)	Microtubule dynamics inhibitor	Breast cancer, liposarcoma	FDA Approved	143
Brentuximab vedotin	*Dolabella auricularia* (sea hare - via dolastatin 10)	Antibody–drug conjugate targeting CD30	Hodgkin lymphoma, ALCL	FDA Approved	144
Polatuzumab vedotin	Derived from dolastatin analogs	Anti-CD79b antibody–drug conjugate	Diffuse large B-cell lymphoma	FDA Approved	145
Marizomib (Salinosporamide A)	*Salinispora tropica* (marine actinobacterium)	Proteasome inhibitor	Multiple myeloma, glioblastoma	Phase III (Clinical)	146
Aurilide analogs	*Dolabella auricularia*	Induces apoptosis via the mitochondrial pathway	Lung, breast, and pancreatic	Preclinical	147

ALCL: Anaplastic Large Cell Lymphoma; CD30: Cluster of Differentiation 30 (a cell surface receptor); CD79b: Cluster of Differentiation 79b (B-cell receptor component); DLBCL: Diffuse Large B-Cell Lymphoma; FDA: Food and Drug Administration.

## Role of emerging technologies in marine research

The integration of emerging technologies such as artificial intelligence (AI), clustered regularly interspaced short palindromic repeats (CRISPR) gene editing, and bioengineering is revolutionizing the landscape of marine drug discovery. AI-powered algorithms are increasingly used to predict bioactivity, toxicity, and drug-likeness of marine-derived compounds, thereby streamlining virtual screening and reducing the time and cost associated with traditional wet-lab testing. These computational tools can rapidly analyze massive datasets, prioritize promising leads, and even suggest novel analogs for synthesis. Meanwhile, CRISPR-Cas9 technology is enabling precise manipulation of biosynthetic gene clusters in marine microorganisms, allowing researchers to enhance or modify the production of desired metabolites, silence competing pathways, and uncover cryptic compounds that are not expressed under standard conditions. In parallel, bioengineering approaches, including metabolic pathway optimization and heterologous expression systems, facilitate the large-scale production of marine bioactives by transferring complex biosynthetic genes into more tractable microbial hosts like *E. coli* or yeast. These innovations not only accelerate the pace of discovery and development but also address critical challenges such as low natural yields and sustainability, thereby paving the way for the clinical translation of novel marine-derived anticancer agents.^148^

## Marine drug development & challenges

Despite the immense therapeutic promise of marine-derived compounds, the path from ocean to clinic is fraught with several significant challenges. One of the primary hurdles is sustainable sourcing, as many marine organisms that produce bioactive compounds exist in fragile ecosystems or are found in limited quantities. Overharvesting could threaten biodiversity and ecological balance, making sustainability a major concern. Additionally, the structural complexity of many marine natural products poses difficulties for large-scale extraction and chemical synthesis, often requiring elaborate, multi-step procedures that are time-consuming, costly, and yield low quantities. Regulatory barriers further complicate development, with stringent guidelines related to marine bioprospecting, environmental impact assessments, and clinical safety evaluations slowing progress. Researchers are turning to innovative strategies such as synthetic biology to overcome these issues, where biosynthetic gene clusters from marine organisms are inserted into easily culturable hosts like *E. coli* or yeast. This enables scalable and eco-friendly production of complex marine compounds in laboratory settings. Aquaculture-based production systems, including cultivating marine sponges, algae, and cyanobacteria under controlled conditions, offer another viable solution to address sustainability and supply challenges. Furthermore, advances in computational drug design and metagenomics are helping to uncover novel bioactive molecules without the need for physically harvesting marine organisms. By integrating these modern technologies with traditional marine pharmacology, the field can more efficiently harness marine biodiversity for drug development while minimizing ecological impact.^149^

## Sustainable sourcing and environmental considerations in marine drug discovery

While the ocean offers a vast and largely untapped resource for novel anticancer compounds, the sustainable sourcing of these marine bioactives presents a major challenge in drug development. Many marine organisms, such as sponges, tunicates, and soft corals, produce bioactive compounds in minute quantities, making large-scale harvesting ecologically harmful and economically impractical. Overexploitation of these species can disrupt marine ecosystems and biodiversity, especially in fragile habitats like coral reefs and deep-sea environments. Synthetic biology and marine biotechnology have emerged as promising alternatives to mitigate these concerns. By identifying and cloning the biosynthetic gene clusters (BGCs) responsible for the production of key compounds—such as polyketides and nonribosomal peptides—researchers can express these genes in fast-growing and sustainable microbial hosts like *Escherichia coli* or Streptomyces species. This approach facilitates scalable production and enables structural optimization through pathway engineering.^150^

Moreover, marine microbial fermentation and aquaculture-based production systems are being explored to cultivate bioactive compound-producing organisms sustainably. For example, *in vitro* cultivation of sponge cells or their symbiotic microbes under controlled bioreactor conditions can offer a renewable supply of compounds without damaging natural populations. Aquaculture of tunicates, algae, and cyanobacteria has also shown promise in producing bulk biomass for compound extraction. Additionally, environmental DNA (eDNA) screening and metagenomic analysis now allows researchers to access biosynthetic potential from uncultivable marine microorganisms, thus expanding the scope of marine drug discovery without the need for extensive organism collection. Despite these advances, regulatory frameworks for marine bioprospecting need to be strengthened to ensure equitable sharing of resources and protection of biodiversity under international agreements such as the Nagoya Protocol.^151^

Altogether, integrating biotechnological innovation with conservation principles is essential to ensure that marine-derived cancer therapeutics are developed environmentally and sustainably.

## Future prospects

It is possible to identify the gene clusters involved in the pathways for the creation of these naturally occurring compounds by sequencing the genome of a microbe that has been found to be a powerful producer of bioactive compounds. Secondary metabolites such as rapamycin, erythromycin, tetracycline, lovastatin, and resveratrol are produced by polyketide synthases (PKSs). Polyketide-producing genes from bacteria and fungi have been cloned, sequenced, and expressed in several hosts. Some marine sponge-associated bacteria that have been found to have the PKSs gene cluster and have antibacterial qualities are being studied. Deep-sea Unusual bioactive compounds are also known to be produced by hydrothermal vent bacteria. Genomic libraries can be created by sequencing the genomes of cultivable microbes and chromosomal DNA.^152^ It is possible to directly separate large genomic DNA fragments from the material and clone them into appropriate host vector systems. The metabolic machinery of a microflora will be available for further manipulation and comprehension through the establishment of comprehensive gene libraries that represent the maximal genome sequences of the sample.

Finding the organism in a symbiotic system that produces metabolites has proven difficult. Attempts to separate different cell types using centrifugation, sieving, or fluorescence-activated cell sorting, and then chemically screen these samples for relevant metabolites, have not been successful. For instance, studies of the D. herbacea–cyanobacterium Oscillatoria spongellae symbiosis have repeatedly confirmed metabolic patterns consistent with those seen in pure cyanobacterial strains.^152^ The cyanobacterial cells were discovered to contain several highly distinctive chlorinated peptides, which had previously been recovered from investigations involving the entire sponge and had a strong structural antecedent in metabolites derived from the free-living cyanobacterium Lyngbya majuscula. However, an analogous approach employs the tunicate Lissoclinum patella. It hosts multiple Prochloron spp. Cyanobacteria produced inconsistent results for several unique cyclic peptides that were discovered to be connected with both tunicate and cyanobacterial cells^153^.

Earlier approaches that mainly screened crude extracts for biological activity have allowed for a plentiful harvest of “low-hanging fruit”. There are now 313 widely used prefractionation techniques and extensive collections of biological assays. Extremely efficient mass spectrometry and nuclear magnetic resonance techniques that are ideal for deciphering complicated structures and synthesis methods on vanishingly small amounts of a chemical. With proper financing, the involvement of academics from other fields, and technical developments, we might expect an even more plentiful crop of new clinical drugs from the sea in the next decade. Nonetheless, a handful of compounds derived from marine sources are already approved; most of these are “first-in-class” drugs, and five of these seven were approved in recent years. Additionally, many marine compounds in preclinical and clinical settings suggest that they may find continued application in human health. Some of these gene clusters have been creatively altered and reengineered. Our knowledge of how these agents are biosynthetically constructed has advanced recently, especially through interdisciplinary techniques, and we are already creating innovative agents with better pharmacological characteristics than the natural product. There are now three MNPs available for purchase, 22 MNPs in clinical trials, and an estimated 118 MNPs under preclinical testing.^154^

As we continue to refine techniques such as fluorescence-activated cell sorting and metabolomic profiling to distinguish metabolite-producing symbionts—e.g., the cyanobacterium Oscillatoria spongellae or Prochloron spp.—from host cells in complex marine systems, drug development pipelines will become increasingly precise and efficient. With three marine-derived drugs currently marketed, 22 in clinical trials, and over 100 under preclinical investigation, the integration of next-gen delivery systems stands to significantly improve the therapeutic index and clinical success of these agents.

Thus, the future of marine-derived anticancer therapy lies with the intersection of marine biosciences, nanotechnology, synthetic biology, and precision drug delivery. Strategic investment in interdisciplinary research and scalable production technologies—such as aquaculture and synthetic platforms—will be crucial to unlock the full potential of marine ecosystems as a prolific source of next-generation cancer therapeutics.

### Limitations of the study

• Limited clinical evidence is available for numerous marine-derived compounds, which restricts their immediate therapeutic translation.
• The exact mechanisms of action for several bioactives remain insufficiently explored.
• Challenges in sustainable sourcing and large-scale production hinder further development and validation.

## Conclusion

In summary, the exploration of marine-based drugs has enormous promise for the development of oncology. The unique chemical variety of marine animals provides a multitude of bioactive compounds with potent anticancer properties. Through extensive research, several chemicals derived from marine sources have demonstrated efficacy in preclinical and clinical settings, suggesting that they may be able to close large therapeutic gaps and enhance existing cancer treatments.

We can better harness these chemicals' therapeutic potential as we gain deeper insights into their mechanisms, such as triggering apoptosis, preventing metastasis, and interfering with cellular signaling pathways. However, there are many obstacles to clinical application, such as the requirement for comprehensive clinical validation, complicated extraction procedures, and sustainable supply methods. Collaboration between biologists, chemists, and oncologists is crucial to maximizing the advantages of marine pharmacology. Furthermore, new developments in synthetic biology and biotechnology may make producing and modifying these substances easier, increasing their accessibility for medical applications.

In the end, as the area develops further, marine-based medications could open the door for personalized medicine catered to each patient's unique characteristics while broadening the range of treatments for different forms of cancer. By embracing the promise of marine biodiversity, we can greatly improve our ability to fight cancer, giving patients all over the world hope and better results.

## Authors contribution

Yash Pramod Patil & Sumit Dilip Ekghara: data analysis, figure preparation, and manuscript writing; Nagaraju Bandaru, Mohan Gandhi Bonthu. Data analysis, supervision, review, and editing; Kunal Sharad Patil & Nagaraju Bandaru: supervision, review, and editing; Nagaraju Bandaru & Sumit Dilip Ekghara: supervision, data discussion, interpretation, critical manuscript revision and editing, project administration, and funding acquisition; Nagaraju Bandaru, Mohan Gandhi Bonthu: review and editing. All the authors have read and approved the final paper.

## Ethics statement

None.

## Data availability statement

The datasets used in the current study are available from the corresponding author on reasonable request.

## Declaration of Generative AI and AI-assisted technologies in the writing process

The authors declare that during the preparation of this work, ChatGPT (OpenAI) was used to assist with language editing, content organization, and summarization of scientific literature. After using this tool, the authors reviewed and edited the content as needed and take full responsibility for the content of the publication.

## Funding

None.

## Conflict of interest

The authors declare that they have no known competing financial interests or personal relationships that could have appeared to influence the work reported in this paper.
